# Rutin Ameliorates ALS Pathology by Reducing SOD1 Aggregation and Neuroinflammation in an SOD1-G93A Mouse Model

**DOI:** 10.3390/ijms251910392

**Published:** 2024-09-27

**Authors:** Xiaoyu Du, Quanxiu Dong, Jie Zhu, Lingjie Li, Xiaolin Yu, Ruitian Liu

**Affiliations:** 1 National Key Laboratory of Biochemical Engineering, Institute of Process Engineering, Chinese Academy of Sciences, Beijing 100190, China; xydu19@ipe.ac.cn (X.D.);; 2 University of Chinese Academy of Sciences, Beijing 100049, China

**Keywords:** amyotrophic lateral sclerosis, rutin, SOD1, neuroprotection, neuroinflammation

## Abstract

Amyotrophic lateral sclerosis (ALS) is a neurodegenerative disorder characterized by the progressive loss of motor neurons, with limited effective treatments. Recently, the exploration of natural products has unveiled their potential in exerting neuroprotective effects, offering a promising avenue for ALS therapy. In this study, the therapeutic effects of rutin, a natural flavonoid glycoside with neuroprotective properties, were evaluated in a superoxide dismutase 1 (SOD1)-G93A mouse model of ALS. We showed that rutin reduced the level of SOD1 aggregation and diminished glial cell activation in spinal cords and brainstems, resulting in significantly improved motor function and motor neuron restoration in SOD1-G93A mice. Our findings indicated that rutin’s multi-targeted approach to SOD1-related pathology makes it a promising candidate for the treatment of ALS.

## 1. Introduction

Amyotrophic lateral sclerosis (ALS) is a neurodegenerative disorder that affects motor neurons, characterized by the progressive degeneration of upper motor neurons (UMNs) located in the motor cortex and brainstem and lower motor neurons (LMNs) situated in the spinal cord’s ventral horn [1]. The exact etiology of ALS remains elusive, but it is known to involve an interplay of genetic, environmental, and lifestyle factors. The majority of ALS patients succumb to respiratory failure within 3 to 5 years post-onset due to the destruction of the neurons regulating the respiratory muscles [2]. This underscores the urgency and significance of developing effective therapeutic drugs for ALS.

Superoxide dismutase 1 (SOD1) is an antioxidant enzyme widely present in cells, and its mutations are closely associated with familial ALS. The mutations in SOD1 were among the earliest identified genetic mutations associated with ALS [3]. These mutations have been extensively studied over time and are well documented as a significant cause of familial ALS, with more than 230 distinct mutations reported to date [4]. The mutations in SOD1 lead to neurotoxicity through various mechanisms, including protein misfolding, proteasome impairment, excitotoxicity, oxidative stress, endoplasmic reticulum stress, impaired axonal transport, inflammation, and mitochondrial dysfunction [5]. Importantly, mutant SOD1 is prone to aggregating in cells. Among various forms of SOD1 aggregates, SOD1 oligomers are proven to be the most neurotoxic and contribute to ALS neuropathology [6,7].

Currently, there are several therapeutic drugs for ALS available globally. These include riluzole, which targets the glutamate excitotoxicity occurring in motor neurons; edaravone, an agent that scavenges free radicals; the histone deacetylase (HDAC) inhibitors and GPBAR1 agonists sodium phenylbutyrate and tauroursodeoxycholic acid; and tofersen, an antisense oligonucleotide that targets SOD1 [8,9,10,11]. However, the therapeutic effects and their scope of application for ALS patients are still limited. As ALS is a multifactorial neurodegenerative disease, it is possible that drugs targeting a single molecular pathway may not result in a complete cure. The combination of multiple drugs or drugs that target multiple therapeutic targets may prove to be a promising avenue for research.

Some natural plant molecules, particularly those belonging to the polyphenol class, can modulate multiple molecular targets associated with neurodegeneration. Rutin, a natural flavonoid glycoside, possesses a variety of biological activities such as antimicrobial, anticancer, antithrombotic, cardioprotective, and neuroprotective effects [12]. These biological functions are primarily related to its anti-inflammatory and antioxidant activities. As a neuroprotective agent, rutin has shown positive effects on various neurodegenerative diseases, including Alzheimer’s disease (AD), Parkinson’s disease (PD), and Huntington’s disease (HD) [13]. In rodent models of AD, rutin has been found to mitigate cognitive deficits by decreasing Aβ aggregation, oxidative stress, and neuroinflammation [14,15,16]. Rutin has been shown to ameliorate the neurotoxicity induced by 6-hydroxydopamine (6-OHDA) in cells and exert protective effects in a PD mouse model involving 6-OHDA [17,18]. Additionally, rutin exhibits antioxidant properties and regulates protein homeostasis, contributing to its neuroprotective role, in transgenic *Caenorhabditis elegans* models of HD [19,20].

Beyond the aforementioned neurodegenerative diseases, rutin has been shown to effectively alleviate the pathological conditions associated with ALS, such as oxidative stress, neuroinflammation, mitochondrial dysfunction, impaired axonal transport, and protein aggregation [21]. A study was conducted to investigate the structural aspects of rutin’s ability to inhibit the aggregation of SOD1 [22]. These clues implicated that rutin may represent a viable therapeutic option for the treatment of ALS, although its effects on an ALS animal model are still undiscovered. In our previous studies, we reported that rutin has therapeutic effects on various amyloid pathologies, such as Aβ and tau, by reducing the formation of toxic aggregates and exhibiting anti-inflammatory properties [14,23,24]. In this study, we investigated the effects of rutin on SOD1 aggregation and cytotoxicity and explored its impact on SOD1 pathology and anti-inflammatory effects in an SOD1-G93A mouse model.

## 2. Results

### 2.1. Rutin Inhibited SOD1-Induced Cytotoxicity

**Figure 1:**
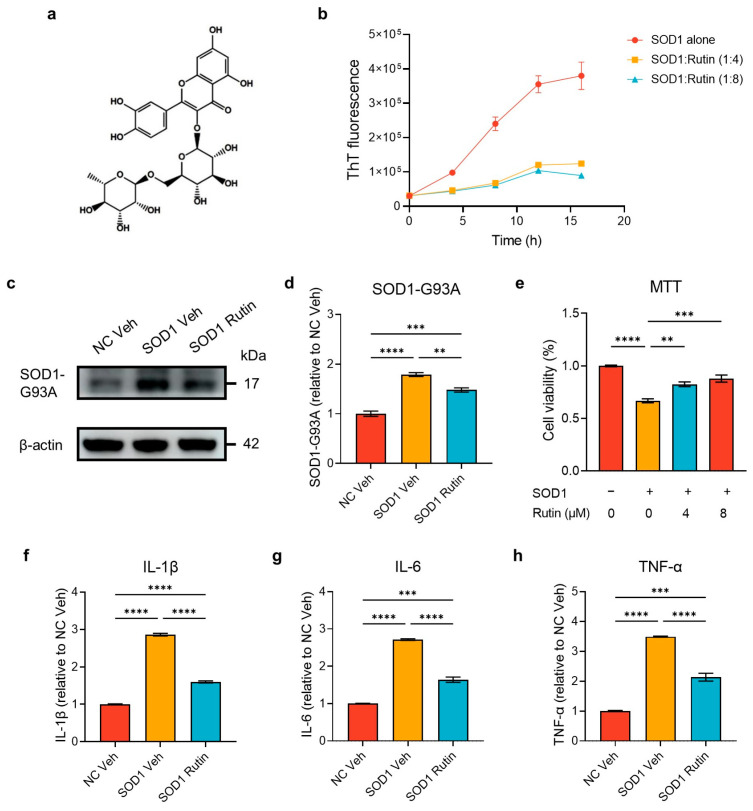
Rutin reduced superoxide dismutase 1 (SOD1)-induced cytotoxicity and neuroinflammation. (**a**) The structural formula of rutin. (**b**) The aggregation kinetics of G93A SOD1. The aggregation state of 50 μM of SOD1 was assessed by a Thioflavin T (ThT) fluorescence assay with or without the incubation of 200 or 400 μM of rutin. (**c**) A Western blot analysis of SOD1 in NSC-34 cells. The cells were transfected with an empty vector (NC-Veh) or a plasmid expressing G93A SOD1 and treated with 8 μM of rutin (SOD1 Rutin) or the vehicle (SOD1 Veh). The SOD1 levels in the cells were analyzed using a Western blot 72 h after transfection. (**d**) Quantification of SOD1 levels in (**b**); *n* = 3 independent experiments. (**e**) The viability of the NSC-34 cells, determined by an MTT assay. The cells were challenged with 1 μM of SOD1 oligomer or PBS, then the cells were treated with 4 μM or 8 μM of rutin or the vehicle; *n* = 3 independent experiments. (**f**–**h**) Inflammatory cytokine levels in primary microglial culture medium. The primary microglia were treated with 1 μM of SOD1 oligomers in the presence (SOD1 Rutin) or absence (SOD1 Veh) of 8 μM of rutin or treated with PBS (NC Veh) for 48 h, then the levels of IL-1β (**f**), IL-6 (**g**), and TNF-α (**h**) in the culture supernatants were measured by an ELISA; *n* = 3 independent experiments. The data represent the mean ± SEM and were analyzed by a one-way ANOVA followed by a Tukey’s test (**d**–**h**). **, *p* < 0.01; ***, *p* < 0.001; and ****, *p* < 0.0001.

Rutin is a flavonoid compound featuring a quercetin molecule glycosidically linked to a rutinose sugar (Figure 1a). To assess the impact of rutin on SOD1 aggregation, we employed the Thioflavin T (ThT) fluorescence assay to monitor the aggregation of SOD1 in the presence or absence of rutin. The results indicated that rutin inhibited the aggregation of the SOD1 protein (Figure 1b). To explore the effects of rutin on the level of intracellular SOD1, we transfected NSC-34 cells with plasmids expressing G93A mutant SOD1 and then the cells were treated with or without rutin. A Western blot analysis demonstrated that the levels of SOD1 were significantly increased in the NSC-34 cells that were transfected with the G93A-SOD1 expression plasmids for 72 h. The rutin treatment resulted in a significantly decreased level of SOD1 in the cells (Figure 1c,d). Subsequently, we assessed the impact of exogenous SOD1 oligomers on the viability of the NSC-34 cells using an MTT assay. The introduction of SOD1 oligomers significantly decreased cell viability, while rutin increased cell viability in a concentration-dependent manner (Figure 1e), suggesting rutin can inhibit SOD1-induced cytotoxicity.

### 2.2. Rutin Mitigated Neuroinflammation Induced by SOD1 Oligomers

We then explored the inhibitory effect of rutin on neuroinflammation. SOD1 oligomers were added to the culture medium of primary microglia in the presence or absence of rutin and incubated for 48 h. Subsequently, we measured the levels of the pro-inflammatory cytokines IL-1β, IL-6, and TNF-α produced by the microglia. The results demonstrated that rutin significantly reduced the levels of the pro-inflammatory cytokines produced by the microglia (Figure 1f–h). This indicates that rutin has the potential to mitigate neuroinflammation by inhibiting the production of pro-inflammatory cytokines.

### 2.3. Rutin Rescued Weight Loss and Motor Impairment in SOD1-G93A Mice

**Figure 2:**
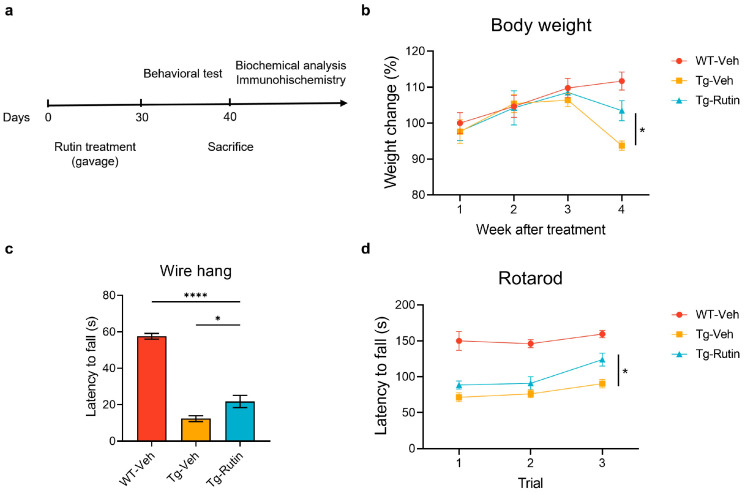
Rutin rescued motor dysfunction in SOD1-G93A mice. (**a**) Schematic diagram of pharmacological treatments and experimental measurements in SOD1-G93A mice. (**b**) The body weights of SOD1-G93A mice treated with (Tg-Rutin) or without rutin (Tg-Veh) and their WT littermates treated with the vehicle (WT-Veh) monitored weekly from 1 to 4 weeks after the treatment; *n* = 8 independent animals. (**c**) The latency to fall of mice during the wire hang test; *n* = 8 independent animals. (**d**) The latency to fall of mice during the rotarod test; *n* = 8 independent animals. The data represent the mean ± SEM and were analyzed by a one-way ANOVA followed by a Tukey’s test (**c**) or a two-way ANOVA followed by a Tukey’s test (**b**,**d**). *, *p* < 0.05; and ****, *p* < 0.0001.

Weight loss due to muscle atrophy, as well as motor dysfunction such as muscle weakness, are the primary symptoms of ALS [1]. To investigate the effect of rutin on body weight and motor function in SOD1-G93A mice, the SOD1-G93A mice were orally administered with rutin once daily for 30 consecutive days (Figure 2a). The body weight of the mice was monitored weekly, and the result revealed that rutin significantly inhibited the decrease in body weight of the SOD1-G93A mice (Figure 2b). Subsequently, the motor function of the mice was evaluated using the wire hang test and rotarod test. The treatment with rutin revealed a significantly increased muscle strength in the SOD1-G93A mice compared to the vehicle control group in the wire hang test (Figure 2c). In the rotarod test, the latency to fall was significantly increased in the SOD1-G93A mice treated with rutin when compared with the vehicle-treated mouse controls, suggesting that rutin significantly ameliorated motor impairment in SOD1-G93A mice (Figure 2d).

### 2.4. Rutin Attenuated SOD1 Pathology in SOD1-G93A Mice

**Figure 3:**
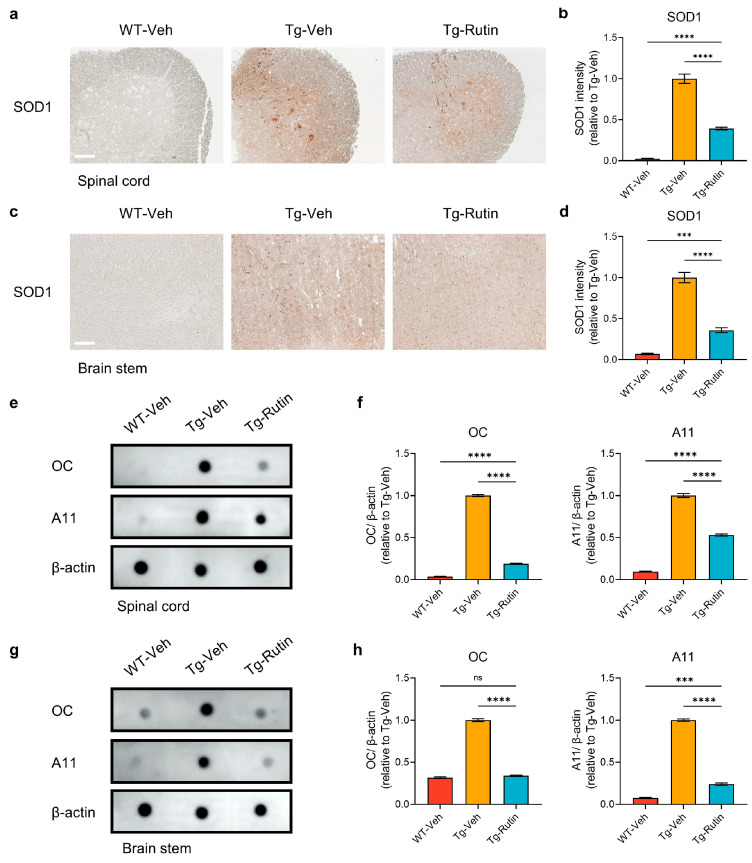
Rutin attenuated SOD1 pathology in SOD1-G93A mice. (**a**) SOD1 immunostaining in the spinal cord of SOD1-G93A mice treated with rutin (Tg-Rutin) or the vehicle (Tg-Veh) and their WT littermates treated with the vehicle (WT-Veh) (scale bar: 60 μm). (**b**) Quantification of SOD1 immunostaining in the spinal cord; *n* = 5 independent animals. (**c**) SOD1 immunostaining in the brainstem of SOD1-G93A mice and their WT littermates treated with rutin or the vehicle (scale bar: 60 μm). (**d**) Quantification of SOD1 immunostaining in the brainstem; *n* = 5 independent animals. (**e**) Dot blot analysis of OC-positive fibrillar SOD1 and A11-positive oligomeric SOD1 in the spinal cord of SOD1-G93A mice and their WT littermates treated with rutin or the vehicle. (**f**) Quantification of OC-positive and A11-positive SOD1 in the spinal cord; *n* = 5 independent animals. (**g**) Dot blot analysis of OC-positive fibrillar SOD1 and A11-positive oligomeric SOD1 in the brainstem of SOD1-G93A mice and their WT littermates treated with rutin or the vehicle. (**h**) Quantification of OC-positive and A11-positive SOD1 in the brainstem; *n* = 5 independent animals. The data represent the mean ± SEM and were analyzed by a one-way ANOVA followed by a Tukey’s test (**b**,**d**,**f**,**h**). ***, *p* < 0.001; ****, *p* < 0.0001; and ns, not significant.

Under pathological conditions, SOD1 abnormally accumulates and forms aggregates, exerting toxic effects [25]. In the ventral horn of the spinal cord and the brainstem of the SOD1-G93A mice, there was a significant presence of SOD1 aggregates detected by SOD1 immunostaining, whereas the rutin treatment significantly reduced the level of the SOD1 aggregates (Figure 3a–d).

Furthermore, we detected SOD1 aggregates in the spinal cord and brainstem of the SOD1-G93A mice using antibodies OC and A11, which specifically recognize amyloid fibrillar oligomers and pre-fibrillar oligomers, respectively. The results showed that rutin treatment reduced the levels of OC-positive SOD1 oligomers by 81.1% and A11-positive SOD1 oligomers by 46.9% in the spinal cord (Figure 3e,f). Similarly, the treatment with rutin resulted in a 66.0% reduction in the levels of OC-positive SOD1 aggregates and a 76.0% reduction in the A11-positive SOD1 oligomer levels in the brainstem, compared with the vehicle-treated SOD1-G93A mice (Figure 3g,h). These results indicated that rutin significantly decreased the levels of SOD1 aggregates in SOD1-G93A mice, which may contribute to the prevention of the SOD1 pathology.

### 2.5. Rutin Alleviated the Depletion of Motor Neurons in SOD1-G93A Mice

**Figure 4:**
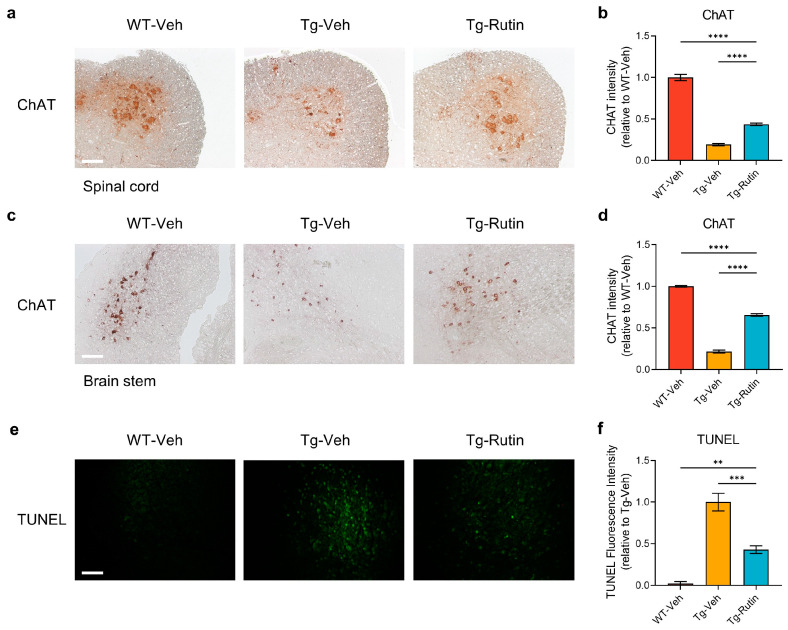
Rutin ameliorated the loss of motor neurons in SOD1-G93A mice. (**a**) Choline acetyltransferase (ChAT) immunostaining in the spinal cord of SOD1-G93A mice treated with rutin (Tg-Rutin) or the vehicle (Tg-Veh) and their WT littermates treated with the vehicle (WT-Veh) (scale bar: 60 μm). (**b**) Quantification of ChAT immunostaining in the spinal cord; *n* = 5 independent animals. (**c**) ChAT immunostaining in the brainstem of SOD1-G93A mice and their WT littermates treated with rutin or the vehicle (scale bar: 60 μm). (**d**) Quantification of ChAT immunostaining in the brainstem; *n* = 5 independent animals. (**e**) TUNEL analysis performed in the spinal cord of SOD1-G93A mice and their WT littermates (scale bar: 40 μm). (**f**) Quantification of TUNEL fluorescence intensity in the spinal cord; *n* = 5 independent animals. The data represent the mean ± SEM and were analyzed by a one-way ANOVA followed by a Tukey’s test (**b**,**d**,**f**). **, *p* < 0.01; ***, *p* < 0.001; ****, *p* < 0.0001.

One of the neuropathological hallmarks of ALS is the loss of motor neurons. We assessed the number of motor neurons in the ventral horn of the spinal cord and the brainstem of the SOD1-G93A mice. Th immunohistochemical results revealed that the SOD1-G93A mice exhibited a significant reduction in the levels of choline acetyltransferase (ChAT), a motor neuron marker in the spinal cord and brainstem, indicating a substantial loss of motor neurons. The treatment with rutin significantly restored the levels of ChAT in the spinal cord and brainstem (Figure 4a–d). Furthermore, we utilized the TUNEL assay to analyze the occurrence of apoptosis in the spinal cord of the mice. The results indicated that the treatment with rutin reduced the level of apoptosis in the spinal cord of the SOD1-G93A mice (Figure 4e,f).

### 2.6. Rutin Reduced Neuroinflammation in SOD1-G93A Mice

**Figure 5:**
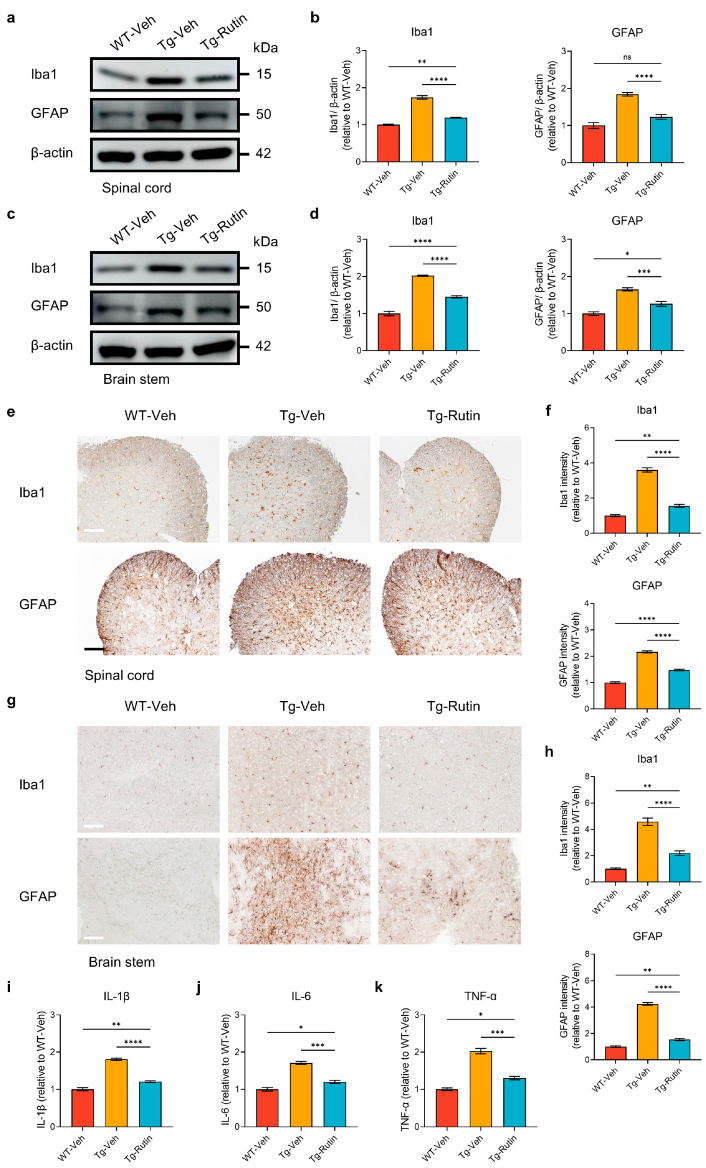
Rutin reduced neuroinflammation in SOD1-G93A mice. (**a**) Western blot analysis of Iba1 and GFAP in the spinal cord of SOD1-G93A mice treated with rutin (Tg-Rutin) or the vehicle (Tg-Veh) and their WT littermates treated with the vehicle (WT-Veh). β-actin was used as the control. (**b**) Quantification of Iba1 and GFAP levels in the spinal cord; *n* = 5 independent animals. (**c**) Western blot analysis of Iba1 and GFAP in the brainstem of SOD1-G93A mice and their WT littermates treated with rutin or the vehicle. β-actin was used as the control. (**d**) Quantification of Iba1 and GFAP levels in the brainstem; *n* = 5 independent animals. (**e**) Iba1 and GFAP immunostaining in the spinal cord of SOD1-G93A mice and their WT littermates treated with rutin or the vehicle (scale bar: 60 μm). (**f**) Quantification of Iba1 and GFAP intensities in the spinal cord; *n* = 5 independent animals. (**g**) Iba1 and GFAP immunostaining in the brainstem of SOD1-G93A mice and their WT littermates treated with rutin or the vehicle (scale bar: 60 μm). (**h**) Quantification of Iba1 and GFAP intensities in the brainstem; *n* = 5 independent animals. (**i**–**k**) Inflammatory cytokine levels in the spinal cord of the mice. The levels of IL-1β (**i**), IL-6 (**j**), and TNF-α (**k**) in the spinal cord of SOD1-G93A mice and their WT littermates treated with rutin or the vehicle were measured by an ELISA; *n* = 5 independent animals. The data represent the mean ± SEM and were analyzed by a one-way ANOVA followed by a Tukey’s test (**b**,**d**,**f**,**h**–**k**). *, *p* < 0.05; **, *p* < 0.01; ***, *p* < 0.001; ****, *p* < 0.0001; and ns, not significant.

Neuroinflammation plays a significant role in the pathology of ALS, and the activation of the glial cells is intimately linked to neurodegenerative processes [26]. To evaluate the inflammatory-related pathology in the central nervous system of the SOD1-G93A mice, we assessed the proliferation of microglia and astrocytes using Iba1 and GFAP immunostaining. The results demonstrated that the levels of Iba1 and GFAP in the ventral horn of the spinal cord and the brainstem of the SOD1-G93A mice were significantly elevated compared with those in the WT mice, indicating a marked activation of the microglia and astrocytes in the ALS pathology, while the treatment with rutin significantly reduced the proliferation of microglia and astrocytes in the spinal cord and brainstem of the SOD1-G93A mice (Figure 5a–d). Consistent with the immunohistochemical findings, the Western blot analysis revealed that the levels of Iba1 and GFAP in the spinal cord and brainstem of rutin-treated SOD1-G93A mice were significantly lower than those in the vehicle-treated mice (Figure 5e–h). Subsequently, we measured the levels of the pro-inflammatory cytokines IL-1β, IL-6, and TNF-α in the spinal cord of the mice. Compared with the vehicle-treated mice, the levels of IL-1β, IL-6, and TNF-α in the rutin-treated SOD1-G93A mice were significantly reduced (Figure 5i–k). These results suggested that rutin diminishes the activation of the glial cells in the SOD1-G93A mice, thereby ameliorating the inflammatory environment and attenuating the ALS pathology.

## 3. Discussion

ALS primarily affects the UMNs and LMNs that control muscle movement, leading to muscle weakness, atrophy, and, ultimately, the loss of function [27]. The pathogenesis of ALS is complex, involving the interplay of genetic and environmental factors. Although the exact etiology is not fully understood, certain genetic mutations, such as those in the SOD1 gene, are associated with familial ALS [28].

Our findings showed that rutin ameliorated motor deficits and reduced motor neuron loss in SOD1-G93A mice, indicating therapeutic potential for ALS. All these beneficial effects may result from the reduction in SOD1 aggregates in the spinal cord and brainstem following rutin treatment. SOD1 aggregation is a key pathological feature of ALS, which is closely associated with neurotoxicity. In our in vitro study, rutin significantly reduced the level of SOD1 in NSC-34 cells and diminished the cytotoxicity induced by SOD1 oligomers. This discovery was consistent with the previous report of the structural aspects of rutin’s ability to inhibit the aggregation of SOD1 in vitro. Rutin can directly bind to SOD1, stabilizing its dimeric conformation and inhibiting its aggregation [22]. In addition, rutin has also been confirmed to exert inhibitory effects on the aggregation of other amyloid proteins such as Aβ and tau in corresponding animal models. The mechanism by which rutin inhibits amyloid aggregation may involve its aromatic structure binding to the hydrophobic β-sheet secondary structures of amyloid aggregates, thereby disrupting their polymerization properties [24,29].

In addition to its protective effects on inhibiting SOD1 aggregation, rutin is well known as a result of its anti-inflammatory properties. Glial cell activation is a key neuroinflammatory process, which links to neuronal injury in ALS pathology [26]. Rutin treatment significantly reduced the Iba1 and GFAP levels in the spinal cord and brainstem of the SOD1-G93A mice, indicating rutin’s anti-inflammatory activities, such as decreasing microgliosis and astrocytosis. Rutin has been shown to effectively alleviate the oxidative stress induced by amyloid protein aggregation and to downregulate the proliferation of microglia and astrocytes, as well as the levels of nitric oxide (NO) and pro-inflammatory cytokines [14,23,24]. It has been demonstrated that rutin can reduce the levels of reactive oxygen species (ROS) and inhibit the expression of pro-inflammatory cytokines and the activation of NLRP3 inflammasome, effectively reversing spinal cord injury [30]. In another study, rutin was found to improve secondary brain injury following subarachnoid hemorrhage by downregulating RAGE, NF-κB, and inflammatory cytokines [31]. Rutin has also been shown to inhibit pro-inflammatory cytokines by activating SIRT1, thereby combating the inflammation induced by hydrogen peroxide [32]. In addition, rutin promotes neuronal cell survival by activating the AKT signaling cascade and has a significant alleviating effect on ROS-induced oxidative stress [33]. In combination with the multifaceted effects of reducing SOD1 aggregates and cytotoxicity and of anti-inflammation, as well as anti-oxidative stress, rutin presents promising therapeutic potential in the treatment of ALS.

Given the complexity of ALS pathogenesis, a drug targeting a single mechanism is hardly enough to cure such a disease. Rutin, however, has the potential to be part of a combination therapy, synergizing with existing or new drugs to target different aspects of the disease. Investigating how rutin interacts with other compounds and identifying the most effective combination regimens could be a key area for future research. Moreover, exploring the potential impact of rutin on non-SOD1-related ALS subtypes is also a research direction worth attention.

Rutin, a natural flavonoid with a wide range of biological activities, has demonstrated the ability to cross the blood–brain barrier, exerting beneficial effects on the central nervous system [34]. After the intravenous administration of rutin at a dose of 10 mg/kg in rats, the maximum concentrations in the plasma and brain homogenate were (1511.24 ± 46.92) ng/mL and (111.57 ± 12.01) ng/mL, respectively [35]. ALS patients have a total SOD1 concentration in the cerebrospinal fluid (CSF) of approximately 45.3 ng/mL [36]. Although rutin has a relatively low bioavailability, it has been shown to be capable of crossing the blood–brain barrier and reaching a sufficiently high concentration in the CSF to neutralize the pathological SOD1 proteins. In addition, efforts have been made to enhance its therapeutic potential for central nervous system disorders. Chitosan-based nanoparticles for the delivery of rutin have been developed, effectively enhancing its bioavailability and brain-targeting efficiency [35]. It has been reported that a sodium salt form of rutin possesses high water solubility and bioavailability, and it has the capability to enhance the permeability of the blood–brain barrier [16].

In summary, our study demonstrated that rutin can alleviate the level of SOD1, improve motor performance, protect motor neurons, and reduce neuroinflammation in SOD1-G93A mice, making it a potential therapeutic agent candidate for ALS treatment.

## 4. Materials and Methods

### 4.1. Plasmid Construction

The pF155 pCDNA3.1(+) SOD1 G93A plasmid was obtained from MiaoLingBio, Wuhan, China. The empty pCDNA3.1 vector was used as a control. For the recombinant SOD1 protein expression plasmids, sequences of human SOD1 were optimized for codon usage in *E. coli*, and the complete gene synthesis was accomplished by Genewiz, Beijing, China. The gene was then cloned into the pET-28a(+) vector to express the his-tagged SOD1 protein.

### 4.2. Purification and Aggregation of SOD1

The plasmid-expressing His-tagged G93A SOD1 was transformed into BL21 cells. An individual colony from the transformation was inoculated into an LB medium containing 100 μg/mL of ampicillin and cultured overnight at 37 °C. On the second day, the overnight culture was diluted 1:100 into a fresh LB medium and grown for 4 h, after which 0.5 mM of IPTG was added to induce the expression of recombinant SOD1 protein, and the culture was continued overnight at 20 °C. Following the induction, the cells were harvested by centrifugation at 5000× *g* for 10 min at 4 °C. The cell pellet was resuspended in a lysis buffer (50 mM of Tris-HCl at a pH of 7.5, 0.5 M of NaCl, 2 mM of β-mercaptoethanol, 10 mM of imidazole, 1 mg/mL of lysozyme, 0.1 mM of PMSF, and a protease inhibitor cocktail) and subjected to homogenization. The disrupted cell suspension was centrifuged at 13,000× *g* for 10 min at 4 °C, and the supernatant was collected. The supernatant was filtered through a 0.22 µm filter, and then passed through a HisTrap column (GE Healthcare Life Sciences, Marlborough, MA, USA) equilibrated with a buffer (50 mM of sodium phosphate at a pH of 7.6, 0.5 M of NaCl, 2 mM of β-mercaptoethanol, and 10 mM of imidazole). The column was washed with a buffer containing 20 mM of imidazole to remove any unbound proteins. The elution was performed using a linear gradient of 20 to 400 mM of imidazole. The obtained protein was dialyzed overnight in PBS and stored at −20 °C until use. The freshly prepared SOD1 was diluted to 50 μM with PBS containing 50 mM of TCEP hydrochloride, followed by incubation in the absence or presence of rutin at 37 °C with continuous stirring at 200 rpm. The aggregation state of the SOD1 was evaluated using the ThT fluorescence assay.

### 4.3. Thioflavin T Fluorescence Assay

To monitor the aggregation of the SOD1 protein, Thioflavin T (ThT) was dissolved in 50 mM of phosphate buffer (at a pH of 6.5) to a final concentration of 5 μM. The ThT fluorescence intensity of the SOD1 samples was measured using a SpectraMax M5 microplate reader (Molecular Devices, San Jose, CA, USA) set to 442 nm/482 nm (excitation/emission) by adding 10 μL aliquots of the samples to 190 μL of the ThT solution in a 96-well black plate.

### 4.4. MTT Assay

The NSC-34 cells were maintained in a culture medium (DMEM/high glucose, Hyclone, Marlborough, MA, USA) supplemented with 10% fetal bovine serum (FBS) and 1% penicillin/streptomycin, under a humidified atmosphere of 37 °C with 5% CO_2_. The cells were seeded into 96-well plates at a density of approximately 7500 cells per 100 µL of the medium. The plates were incubated at 37 °C for 24 h to allow cell adhesion. The PBS or 1 μm of SOD1 oligomers was added to the culture medium of the cells. Subsequently, different concentrations of rutin were added to the cell cultures and the plates were further incubated for 72 h at 37 °C. The cell viability was assessed by adding 25 µL of 5 mg/mL MTT to each well. After a 3 h incubation at 37 °C, the medium was gently removed and 150 µL of DMSO was added to each well. The plates were then agitated for 10 min at room temperature, and the absorbance at 570/630 nm was measured using a SpectraMax M5 microplate reader (Molecular Devices, San Jose, CA, USA).

### 4.5. Primary Microglia Culture

The primary microglia were obtained from the cerebral cortex of the postnatal mice (P1–P2). In brief, the mouse cerebral cortex was dissected, digested, and triturated. The resulting dissociated cells were cultured in a DMEM/F12 medium with 10% FBS. The loosely attached microglia were harvested at DIV12 by shaking.

### 4.6. ELISA

The levels of IL-1β, IL-6, and TNF-α in the cell culture media and mouse tissue homogenates were measured using ELISA kits (Biolegend, San Diego, CA, USA) according to the manufacturer’s protocol. The absorbance of the reaction was measured at 450 nm using a SpectraMax M5 microplate reader (Molecular Devices, San Jose, CA, USA).

### 4.7. Mice and Rutin Administration

Male 10-week-old SOD1-G93A transgenic mice were originally obtained from the Jackson Laboratory (stock no. 002726) [33]. These mice express the G93A mutant form of the human SOD1 protein. Non-transgenic littermates served as the controls. All the mice used in the experiments were group-housed with free access to food and water and were maintained in a climate-controlled room at a temperature of 22 ± 2 °C and a humidity of 45% ± 10% under a 12 h light/dark cycle. All the experiments were conducted in accordance with the China Public Health Service Guide for the Care and Use of Laboratory Animals. The mouse experiments and protocols were approved by the Institution Animal Care and Use Committee of the Institute of Process Engineering. All the mice were randomly treated in cohorts. Rutin was suspended in 0.5% carboxymethyl cellulose (CMC) to a concentration of 10 mg/mL. All the mice were orally administered rutin (100 mg/kg) or the vehicle (0.5% CMC) daily (100 μL of rutin suspension or the vehicle per 10 g of body weight) for 30 consecutive days. The SOD1-G93A mice were divided into two groups: the rutin group (Tg-rutin) and the vehicle group (Tg-Veh). The wild-type littermates were placed in the vehicle group (WT-Veh). After the final administration, all the mice were tested in the motor function tests.

### 4.8. Wire Hang Test

The mice were placed on the lids of the cages, then the lids were inverted and positioned 50 cm above the bedding material. The latency for each mouse to fall from the lid was observed for up to 60 s. This latency was recorded as the time to fall. Each mouse underwent the test three times, with a one-hour interval between each trial.

### 4.9. Rotarod Test

The motor function of the mice was assessed using a rotarod apparatus (TSE Systems, GmbH, Berlin, Germany). The daily training sessions involved a 5 min acclimation period at a speed of 4 rpm. One hour post-training, the mice underwent three trials of accelerating rotarod tests, with the speed increasing from 0 to 40 rpm over a period of 300 s. Each trial was separated by a 30 min interval. The latency to fall from the rod was recorded. The mice that remained on the rod for a period exceeding 300 s were removed from this study, and their times were recorded as 300 s. The performance of each mouse for that day was expressed as the average of the three trials. The testing was conducted over three consecutive days.

### 4.10. Immunohistochemistry Analysis

The mice were deeply anesthetized using tribromoethanol, and, after cardiac perfusion with cold PBS containing heparin (10 U/mL), they were euthanized. Their brains and spinal cords were immediately excised. The left hemisphere and the spinal cord were fixed overnight in a 4% paraformaldehyde phosphate buffer solution at 4 °C and processed for the paraffin embedding and sectioning. For the immunostaining, 5 μm thick coronal serial sections were deparaffinized and subjected to antigen retrieval in the citrate buffer (0.01 M at a pH of 6.0) for 20 min at 95 °C. The sections were then incubated with 3% H_2_O_2_ and washed three times with PBS. Subsequently, the sections were permeabilized with 10% normal goat serum in 0.3% Triton X-100 PBST for 1 h at room temperature. The sections were incubated with primary antibodies and subsequently with HRP-conjugated secondary antibodies for DAB (3,3′-Diaminobenzidine) immunostaining. The DAB staining was visualized using an Olympus IX73 (Olympus, Tokyo, Japan) inverted microscope with a DP80 camera. The primary antibodies used were as follows: SOD1 (Abcam, Cambridge, UK, ab13498, 1:100), ChAT (Abcam, ab178850, 1:100), Iba1 (GeneTex, Irvine, CA, USA, GTX101495, 1:100), and GFAP (Cell Signaling Technology, Danvers, MA, USA, 3670S, 1:100). For the TUNEL staining, a TUNEL cell apoptosis detection kit (Beyotime, Shanghai, China) was used according to the manufacturer’s protocol. The fluorescence signals were captured using an Olympus IX73 inverted microscope. All the images were analyzed using Image J software (v 1.54f).

### 4.11. Tissue Lysate Preparation and Western Blot

The tissues were homogenized in a modified RIPA buffer containing a complete protease inhibitor cocktail tablet (Sigma, Burlington, MA, USA, P2714-1BTL). The homogenate was then centrifuged at 4 °C at 17,000× *g* for 30 min. The supernatant was collected, and the protein concentration was determined using the BCA protein assay. The protein samples were separated by 4–20% SDS-PAGE gel and transferred onto nitrocellulose membranes. After blocking with 5% non-fat milk for 1 h at room temperature, the membranes were probed with the primary antibodies. Following incubation with the primary antibodies, the membranes were then probed with the corresponding HRP-conjugated secondary antibodies. The bands in the immunoblots were visualized using the Amersham Imager 680 imaging system (GE Healthcare, Marlborough, MA, USA) with enhanced chemiluminescence and quantified by Image J software. The primary antibodies used were as follows: SOD1 (Abcam, ab13498, 1:1000), Iba1 (GeneTex, GTX101495, 1:1000), GFAP (Cell Signaling Technology, 3670S, 1:1000), and β-actin (Beyotime, AF2811, 1:1000).

### 4.12. Dot Blot

The protein samples were applied to nitrocellulose membranes. The membranes were blocked with 5% non-fat milk for 1 h at room temperature, and the membranes were probed with the primary antibodies. Following incubation with the primary antibodies, the membranes were then probed with the corresponding HRP-conjugated secondary antibodies. The blots were visualized using enhanced chemiluminescence with an Amersham Imager 680 imaging system (GE Healthcare) and quantified using Image J software. The primary antibodies used were as follows: A11 (Invitrogen, Waltham, MA, USA, AHB0052, 1:1000), OC (Sigma-Aldrich, Burlington, MA, USA, AB2286, 1:1000), and β-actin (Beyotime, AF2811, 1:1000).

### 4.13. Statistical Analysis

The data were analyzed with GraphPad Prism v.9.5.0. The statistical significance was assessed using a Student’s *t*-test, a one-way ANOVA, or a two-way ANOVA, followed by a Tukey’s test. The results were shown as the group mean ± SEM, and *p* < 0.05 was considered statistically significant.

## 5. Conclusions

Our study demonstrated that rutin can alleviate the level of SOD1, improve motor performance, protect motor neurons, and reduce neuroinflammation in SOD1-G93A mice, making it a potential therapeutic agent candidate for ALS treatment.

## Data Availability

The original contributions presented in this study are included in this article; further inquiries can be directed to the corresponding authors.
